# Extra-pair parentage and personality in a cooperatively breeding bird

**DOI:** 10.1007/s00265-018-2448-z

**Published:** 2018-02-15

**Authors:** Hannah A Edwards, Hannah L Dugdale, David S Richardson, Jan Komdeur, Terry Burke

**Affiliations:** 1 Department of Animal and Plant Sciences, University of Sheffield, Sheffield, S10 2TN UK; 2 School of Biology, The Faculty of Biological Sciences, University of Leeds, Leeds, LS2 9JT UK; 3 Behavioural Ecology and Physiological Group, Groningen Institute for Evolutionary Life Sciences, University of Groningen, PO Box 11103, 9700 cc Groningen, The Netherlands; 4 School of Biological Sciences, University of East Anglia, Norwich Research Park, Norwich, NR4 7TJ UK; 5 Nature Seychelles, PO BOX 1310, Mahe, Republic of Seychelles

**Keywords:** Seychelles warbler, Personality, Exploration, Extra-pair paternity, Reproductive behaviour

## Abstract

**Abstract:**

Why so much variation in extra-pair parentage occurs within and among populations remains unclear. Often the fitness costs and benefits of extra-pair parentage are hypothesised to explain its occurrence; therefore, linking extra-pair parentage with traits such as personality (behavioural traits that can be heritable and affect reproductive behaviour) may help our understanding. Here, we investigate whether reproductive outcomes and success are associated with exploratory behaviour in a natural population of cooperatively breeding Seychelles warblers (*Acrocephalus sechellensis*) on Cousin Island. Exploratory behaviour correlates positively with traits such as risk-taking behaviour and activity in other wild bird species and might promote extra-pair mating by increasing the rate at which potential extra-pair partners are encountered. We therefore predicted that fast-exploring individuals would have more extra-pair offspring. There is also a potential trade-off between pursuing extra-pair parentage and mate guarding in males. We therefore also predicted that fast-exploring males would be more likely to pursue extra-pair parentage and that this would increase the propensity of their mate to gain extra-pair parentage. We found that neither the total number of offspring nor the number of extra-pair offspring were associated with a male’s or female’s exploratory behaviour. However, there was a small but significant propensity for females to have extra-pair fertilisations in pairs that were behaviourally disassortative. Overall, we conclude that, due to the small effect size, the association between exploratory behaviour and extra-pair paternity is unlikely to be biologically relevant.

**Significance statement:**

True genetic monogamy is rare, even in socially monogamous systems, and multiple factors, such as behaviour, social structure, morphology and physiology, determined by the biological system can cause variation in extra-pair parentage (EPP). Therefore, investigating the inherent differences in these factors among individuals could be informative. We investigated whether reproductive outcomes/success are associated with differences in the propensity to explore novel environments/objects in a promiscuous, island-dwelling cooperatively breeding bird, the Seychelles warbler. Our results showed that exploratory behaviour was not associated with the number of offspring produced by an individual, and thus the long-term fitness consequences of different exploratory tendencies did not differ. We also found that the propensity to engage in EPP in females was higher in dissimilar behavioural pairs, but due to the small effect size, we hesitate to conclude that there are personality-dependent mating outcomes in the population.

**Electronic supplementary material:**

The online version of this article (10.1007/s00265-018-2448-z) contains supplementary material, which is available to authorized users.

## Introduction

True genetic monogamy is rare, even in socially monogamous systems (Griffith et al. 2002; Uller and Olsson 2008; Cohas and Allainé 2009). The occurrence of extra-pair parentage (EPP) is often associated with the sex-related trade-offs of engaging in EPP within a population, which can result in sexual conflict over the optimal strategy. For example, EPP will predominantly benefit females if it helps to avoid inbreeding (Arct et al. 2015), has an indirect genetic benefit for offspring or helps to assure fertility (Griffith et al. 2002; Akçay and Roughgarden 2007). However, EPP may also carry costs for both males and females through the loss of foraging opportunities (Rowe 1992), an increased risk of death or injury (Magnhagen 1991; Rowe 1994; Réale et al. 1996; Arnold and Owens 2002; Eliassen and Jørgensen 2014), a reduction in the amount of parental care given by the social mate (Burke et al. 1989; Dixon et al. 1994; Schroeder et al. 2016) and, solely for males, the loss of paternity at the social nest (Petrie and Kempenaers 1998). The extent to which these trade-offs are mediated will be dependent on a suite of environmental, social, morphological, physiological and behavioural factors (Gowaty 1996; Kokko et al. 2006: Clutton-Brock 2007). Therefore, by considering the costs and benefits of EPP in relation to the inherent differences among individuals, we might further our understanding of why variation in EPP occurs (Eliassen and Kokko 2008).

Consistent individual differences in behaviour, termed animal personality, can impact upon the fitness of individuals (e.g. Dingemanse and Réale 2013). However, the relationship between personality and reproductive success is often ambiguous in wild populations and can be under different selection pressures in different contexts (e.g., fluctuations in food availability, Réale and Festa-Bianchet 2003; Dingemanse et al. 2004; Le Cœur et al. 2015), and be affected by the interaction between the personalities within social pairs (Dingemanse et al. 2004; Both et al. 2005; Gabriel and Black 2012; Burtka and Grindstaff 2015). A meta-analysis by Smith and Blumstein (2008) across a range of taxa found evidence for fitness trade-offs associated with certain personality traits. Survival rates for bolder and faster-exploring individuals were lower than for their shyer, slower counterparts, but bolder and aggressive individuals had a higher reproductive success rate (a combination of annual and lifetime success) than their shyer, less aggressive counterparts.

Personality can also explain individual variation in alternative reproductive outcomes, such as EPP (Duckworth 2006; van Oers et al. 2008; While et al. 2009; Patrick et al. 2011; Martin et al. 2014; McCowan et al. 2014). Previous research in socially monogamous species has revealed that the exploratory or aggressive nature of an individual can influence the mode of paternity acquisition. For example, fast-exploring males and aggressive females exhibited high rates of extra-pair parentage (While et al. 2009; Patrick et al. 2011; Martin et al. 2014). Alternatively, the personality of the social partner can strongly affect the reproductive behaviour of the focal individual (Niemelä and Santostefano 2015). For example, in great tits (*Parus major*), within the social pair, the female’s personality affected the probability of the male gaining extra-pair parentage (Patrick et al. 2011). In this case, it was postulated that the high activity levels of fast-exploring females may reduce the time spent at the social nest and, in turn, the male’s certainty of paternity, thereby reducing the pair male’s commitment to the brood and encouraging EPP (Patrick et al. 2011). High similarity between the personalities within the social pair has also been found to correlate with an increased likelihood of being cuckolded (van Oers et al. 2008). Personality, if it is associated with attractiveness (Goddin and Dugatkin 1996) and thus extra-pair potential, may therefore be a mechanism that determines the occurrence of EPP in the social brood (van Oers et al. 2008). Collectively, these studies suggest that the personalities of both members of a pair have the potential to influence parentage, and this is likely to be influenced by the sex-related trade-offs of engaging in EPP within a population (Patrick et al. 2011).

The cooperatively breeding Seychelles warbler (*Acrocephalus sechellensis*) population on Cousin Island offers the novel opportunity to test in a variable social environment the hypothesis that personality traits are associated with EPP. In this population, primary pairs form long-term bonds and jointly hold a breeding territory, but there is a high rate of extra-group paternity, with 44% of offspring being sired by primary males outside of the natal territory (Richardson et al. 2001; Hadfield et al. 2006). Extra-pair fertilisations (EPF) have been shown to be influenced by a male’s major histocompatibility complex (MHC) genotype (Richardson et al. 2005) and thus might provide genetic benefits to females and their offspring (Brouwer et al. 2010). However, EPP is regulated by the social male’s mate guarding the social female (Komdeur et al. 1999, 2007). Behavioural traits that are repeatable and heritable in this study system, and thus cause long-term differences among individuals, are exploration in novel environments and exploration of novel objects (Edwards et al. 2015; Edwards et al. 2017). These exploratory traits have been shown to be positively correlated with risk-taking behaviour (e.g. Quinn et al. 2012), activity (e.g. Quinn and Cresswell 2005) and dispersal (Dingemanse et al. 2003; Korsten et al. 2013) in other wild bird species. Fast exploratory behaviour may therefore result in high encounter rates with potential extra-pair partners; this may be equivalent to the effect of increasing bird density, which has been found to increase the rate of EPP (e.g. Richardson and Burke 2001; Brouwer et al. 2017).

Here, we predict that, due to the potential for fast explorers to increase encounter rates with extra-pair partners, fast-exploring individuals (males and females) will have more extra-pair offspring. However, within the social pair, EPP is regulated by the social male mate guarding the social partner (Komdeur et al. 1999, Komdeur 2001, 2007). We therefore predict that, within the social pair, both sexes in fast-exploring pairs will have more extra-pair offspring. We predict that fast-exploring males will be more likely to encounter extra-pair partners and so trade-off the benefit of gaining paternity through EPP in other nests against the cost of nest guarding, and the higher possibility of being cuckolded. We also predict that fast-exploring females will exploit this trade-off to encounter a high number of extra-pair partners, so helping to potentially gain indirect genetic benefits.

## Methods

### Study system

The Seychelles warbler is a small passerine endemic to the Seychelles. It has a facultative cooperatively breeding system (i.e. individuals may forego reproduction to raise offspring that are not their own (Cockburn 1998)). The study population on Cousin Island (0.29 km^2^; 4°20′S, 55°40′E) consists of ca 320 individuals that are distributed across 110–115 territories (Komdeur and Pels 2005). Primary breeding birds defend a territory year-round and form long-term pair bonds, often until death (mean lifespan 5.5 years from fledgling, Komdeur 1991). Habitat saturation means that breeding opportunities are rare, and individuals are forced to delay independent breeding and may then remain as non-primary breeders within a good-quality (high food abundance) territory (Komdeur 1992). In general, there is a single clutch in a breeding season, consisting of a single egg, although 13% of nests contain two or more eggs (Richardson et al. 2001), with two breeding seasons per year. All nests are followed until failure or fledging (fledgling success is ~ 80%; Komdeur 1994). Non-primaries often (but not always) help raise offspring, and for female non-primaries this decision depends on the continued presence of the primary female that raised them (Richardson et al. 2003). Non-primary females may sometimes gain maternity by laying an egg in a primary female’s nest, but non-primary males rarely gain parentage and extra-pair offspring are primarily fathered by primary breeding males outside of the natal territory (Richardson et al. 2001). Helpers convey long-term survival benefits on the offspring they help (Komdeur 1994; Brouwer et al. 2012).

Seychelles warblers were monitored on Cousin during the winter (Jan–Feb) and summer (Jun–Sep) breeding seasons. During both seasons, territory boundaries were defined and individuals followed for approximately 15 min on a weekly basis to ascertain social status and identify breeding attempts (Richardson et al. 2007). Eye colour, which transitions with age (Komdeur 1991), was used to calculate a continuous measure of age. A primary status was assigned to individuals observed as a pair in a territory over multiple weeks, based on key pair-behaviours, such as close proximity to one another and frequent vocal interactions (Komdeur 1992; Richardson et al. 2002, 2007). A non-primary status was assigned to single birds observed consistently within a territory, that did not express primary pair-behaviour, and that were observed interacting non-antagonistically with group members (Richardson et al. 2002). Mist nets were used to capture individuals, which were then ringed with a combination of three colour and one British Trust for Ornithology (BTO) rings to uniquely identify individuals. Where possible, chicks were also ringed in the nest. Blood samples were collected from all captured birds for molecular sexing (following Griffith et al. 2002) and parentage analyses undertaken, enabling individuals to be assigned a natal territory. Furthermore, to estimate food abundance, 14 locations across the island are sampled annually for insects by estimating the number of invertebrates on the undersides of 50 leaves for each tree species present at each location and extrapolating from this to estimate food abundance according to the amount of foliage at each location during the main breeding season each year (Komdeur 1992).

### Pedigree

We extracted DNA from blood using a modified ammonium acetate protocol (Bruford et al. 1998; Richardson et al. 2001) or, for birds caught from 2013 onwards, using a Qiagen DNeasy blood and tissue kit (Qiagen, Crawley, UK). Alleles were visualised and scored using Genotyper 2.5 or Genemapper 3.7 (Applied Biosystems). Parentage was assigned by analysing genotypes at 30 microsatellite loci (Richardson et al. 2002; Spurgin et al. 2014) in the Bayesian R-package MasterBayes 2.52 (Hadfield et al. 2006) in R 3.2.2 (R Core Team 2016). The numbers of unsampled dams and sires were estimated by MasterBayes in each analysis. Tuning parameters were specified to ensure that the Metropolis–Hasting acceptance rates ranged between 0.2–0.5. To ensure that autocorrelations between successive parameter estimates were < 0.1, the number of iterations was increased to 130,000 with a thinning interval of 100 and a burn-in of 30,000. Both parents were sampled simultaneously in all analyses. The parentage analyses were run in three steps, assigning parentage over all of the years in each analysis. In total, 436 offspring were assigned a mother and 491 were assigned a father with at least 80% confidence (HLD et al. unpublished data).

### Personality assays

Birds were assayed for personality during the summer of 2010 and the winter and summer breeding seasons of 2012–2015, for exploration of a novel environment, and in 2013–2015 for exploration of a novel object. Once a bird was caught in a mist net it was blood sampled and measured for morphometric traits, taken back to the field station, rested for 5 min in a bird bag, assayed for personality and then released back at its territory. Exploration of a novel environment was assayed in an Oxygen 4 tent (L322 × W340 × H210 cm; Gelert Ltd. Wigan in blue or green fabric) containing three artificial trees (following the methods in Edwards et al. 2015, 2016, 2017; and adapted from Verbeek et al. 1994). The number of flights, hops and the number of unique trees visited were recorded during a 5-minute period by observing through a small opening (15.2 cm wide by 6.4 cm tall) in the gauze of the tent door. The numbers of flights, hops and unique trees visited were correlated (*n* = 312, hops and flight: *R* = 0.60, *p* < 0.0001; hops and trees: *R* = 0.64, *p* < 0.0001; flight and trees: *R* = 0.70, *p* < 0.0001). The numbers of hops, flights and unique trees visited were totalled to give a measure of exploration of the novel environment. Novel environment exploration is repeatable in the dataset used for this analysis (*R* = 0.19, credible intervals = 0.01–0.31, and in previous analyses *R* = 0.21, credible intervals = 0.09–0.36, Edwards et al. 2015, 2017).

Exploration of a novel object was assayed 2 min after the exploration assay (to allow time for habituation to the novel environment of the tent; see acclimation test Edwards et al. 2015). A novel pink toy attached to a tree branch (95 cm long) was positioned in the centre of the tent (following the methods in Edwards et al. 2015, 2016, 2017; and adapted from Verbeek et al. 1994). For each bird, we included a control assay in which the novel toy was excluded and the tree branch was inserted into the tent to confirm that the behavioural reaction resulted from the novel toy and not the tree branch to which it was attached. The behaviour score (sum of number of hops, fights and trees visited in 5 min) was higher (Edwards et al. 2015), latency time (seconds to move once the assay had begun) was shorter (Wilcoxon signed rank test; *n* = 185, *V* = 3162, *p* < 0.001), and the number of stick touches was lower (Wilcoxon signed rank test; *n* = 185, *V* = 3162, *p* < 0.001) in the novel object assay than in the control assay (Edwards et al. 2017). Latency had very low repeatability (0.02, 95% credible Interval [CrI] = 0.01–0.36, *n* = 177). Therefore, the number of hops, flights and unique trees visited in a 5-min period was totalled to give a measure of exploration of the novel object. Novel object exploration was repeatable in the dataset used for this analysis (*R* = 0.08, credible intervals = 0.02–0.45, and in previous analyses *R* = 0.37, credible intervals = 0.07–0.59, Edwards et al. 2015, 2017).

It was not possible to record data blind because our study involved focal animals in the field. Personality assays were collected on 168 individuals with paternity data (1 measure = 166, 2 measures = 93, 3 measures = 29, 4 measures = 10, 5 measures = 5, 6 measures = 3) for novel environment exploration and 91 individuals with paternity data (1 measure = 90, 2 measures = 38, 3 measures = 2) for novel object exploration.

### Statistical analyses

All statistical analyses were performed in R 3.0.1. (R Development Core Team 2016) using the MCMCglmm package 2.17 (Hadfield 2009).

#### (i) Individual analyses

For the individual data we tested for the effect of exploratory behaviour on (1) the total number of offspring to which an individual was assigned parentage per season, across multiple years, regardless of the mode of paternity, using a Poisson distribution with log link; (2) the number of offspring that the focal individual gained outside the social pair and within the social pair per season, across multiple years, using a binomial distribution with logit link; and (3) whether an individual had produced/sired extra-pair offspring (EPO, yes/no) per season, across multiple years, with a binomial distribution and logit link. For the Poisson and binomial models, we ran separate models for each sex and with exploration of novel environment (*n* = 171) or exploration of a novel object (*n* = 93) as a fixed effect. All the models contained the following fixed effects: social status (non-primary or primary, Richardson et al. 2001, for the novel object exploration dataset only, as no non-primary males were assigned as fathers in this dataset), the linear and quadratic term of age (defined as the number of breeding seasons from an individual’s birth to when its offspring was born, mean centred and divided by two standard deviations; Gelman and Hill 2006), year of birth, the year of the offspring’s birth, the year of exploratory behaviour assay, and the annual insect abundance (average of the 14 locations sampled during the main breeding season, Kaiser et al. 2015). Both an individual’s first exploration score and mean exploration score gave similar results in our analyses (Spearman’s rank correlation coefficient between the first and mean score in the novel environment exploration assay: *n* = 140, *r* = 0.73, *p* < 0.0001, and the novel object exploration assay: *n* = 40, *r* = 0.87, *p* < 0.0001). We therefore used an individual’s first score to allow us to include tent colour (blue/green) for the novel environment exploration models as a fixed effect (Edwards et al. 2017). Since the presence of helpers in a territory can improve nestling survival and thus the detection of EPP (Komdeur 1994; Brouwer et al. 2012), we also included a seasonal helper variable as a fixed effect. This was calculated as the number of helpers in an offspring’s natal territory in model 1, and as the number of helpers in an offspring’s natal territory divided by the total number of offspring gained in a season in models 2 and 3. Bird identity was included as a random effect to account for repeat observations of birds assigned as parents in more than one season.

#### (ii) Pair similarity analyses

For the social pair data, we tested for the effect of exploratory behaviour on (1) the total number of offspring to which a female was assigned maternity per season, with a Poisson distribution and a log link; (2) the total number of offspring to which a male was assigned paternity per season, with a Poisson distribution and log link; (3) whether an EPO had been produced (yes/no) by the female per season, with a binomial distribution and a logit link; and, (4) whether an EPO had been sired (yes/no) by the male per season, with a binomial distribution and logit link. All models contained the following fixed effects: the linear and quadratic terms of age, year of birth, the year of exploratory behaviour assay, annual insect abundance, the seasonal helper variable, year of the offspring’s birth, the male’s exploration score and the female’s exploration score (for individuals tested more than once, we used the score closest in time to when the pair were in a social pair), and an interaction between the male’s and female’s exploration scores. A quadratic term for the partner’s personality was included to model pair similarity. Personality assay number and tent colour were also included as fixed effects. Bird identity was included as a random factor to account for individuals with more than one social mate in the dataset. We ran models 1–4 with exploration of a novel environment as a fixed effect (numbers of: pairs = 76, males = 64, females = 67). The pair model estimates for the number of EPO to WPO and all of the novel object exploration model estimates were not robust, having failed convergence tests, which we believe was due to sample sizes (numbers of pairs = 31, males = 29, females = 30). We therefore did not include the results in this analysis.

In the individual analysis, we specified an Inverse Wishart (*V* = 1, *n* = 0.2) prior for the Poisson models and the proportion of EPO to WPO binomial models. We also specified *V* = 1 and *n* = 2 for the residual, and an inverse Wishart structure for the random effects in the EPO (yes/no) binomial models. In the pair analysis, we specified a parameter expanded structure (*V* = 1, *n* = 1, alpha.mu = 0, alpha.*V* = 1000) for the Poisson models, and *V* = 1 and *n* = 2 for the residual and a parameter expanded structure (*V* = 1, *n* = 1, alpha.mu = 0, alpha.*V* = 1000) for the random effects in the binomial models. We sampled the posterior distribution every 100 iterations, with a burn-in period of 3000 iterations and a run of 203,000 iterations. Our priors were chosen after assessing convergence by using the heidel.diag and geweke.diag functions, and inspecting the autocorrelation values (*r* < 0.1) and time-series plots. All significant results were corrected for multiple testing by false discovery rate (Benjamini and Hochberg 1995).

##### Data availability

The datasets generated during and/or analysed during the current study are available in the Figshare repository: 10.6084/m9.figshare.5739801.v1

## Results

### Individual analyses

**Fig. 1:**
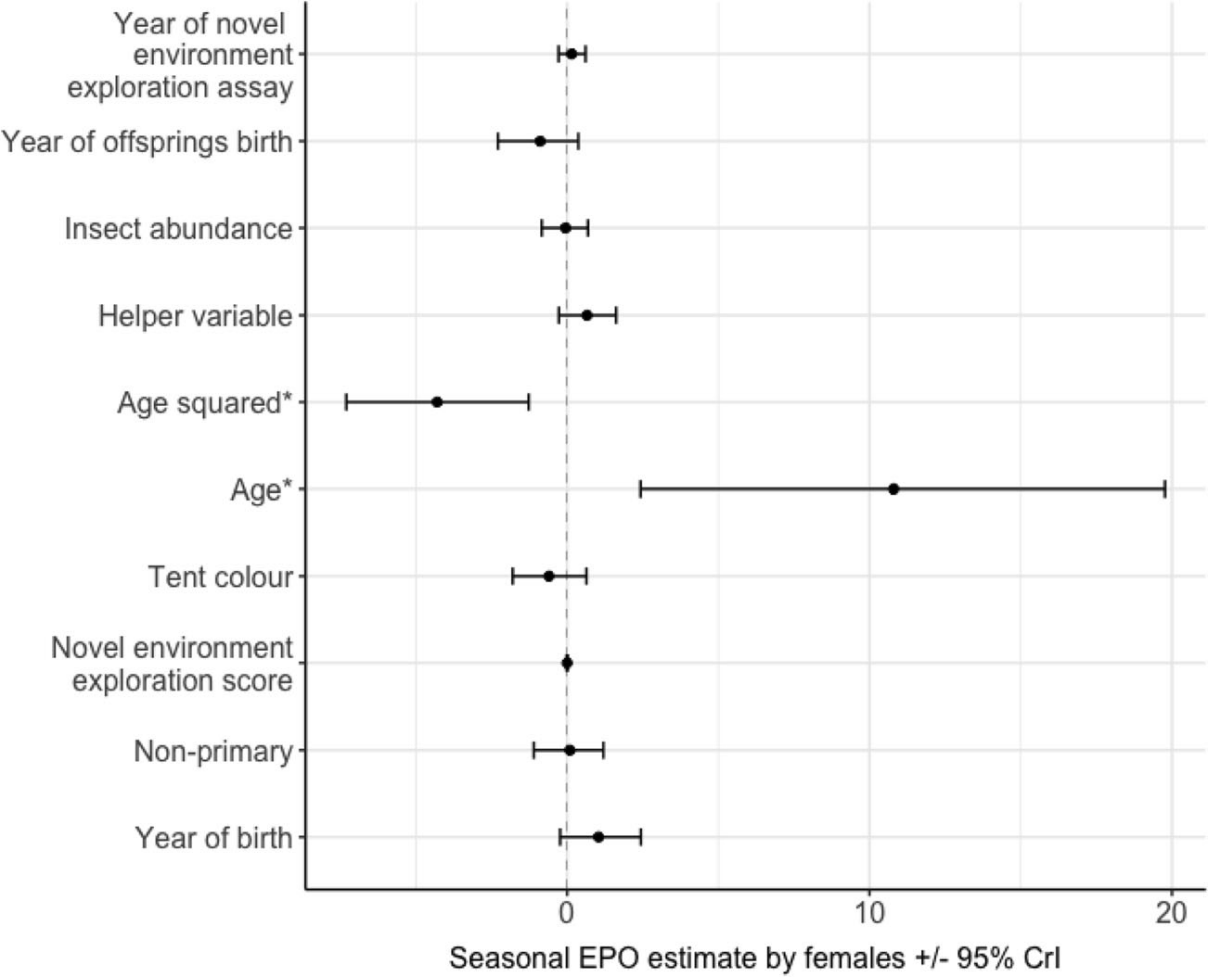
Factors predicting seasonal production of extra-pair offspring (EPO) by females, showing the posterior mode estimates for the fixed effects in the categorical model: year of offspring’s birth, annual insect abundance, helper number (the number of helpers in an offspring’s natal territory), age (quadratic and linear terms)*, tent colour (*N*: blue = 67, green = 18; contrast level = blue), novel environment exploration score, social status (*N*: only primary = 66, only non-primary = 8, assigned offspring as non-primary and as a primary = 11; contrast level = primary) and year of birth. * indicates posterior modes whose 95% credible intervals (CrI) do not overlap zero

**Fig. 2:**
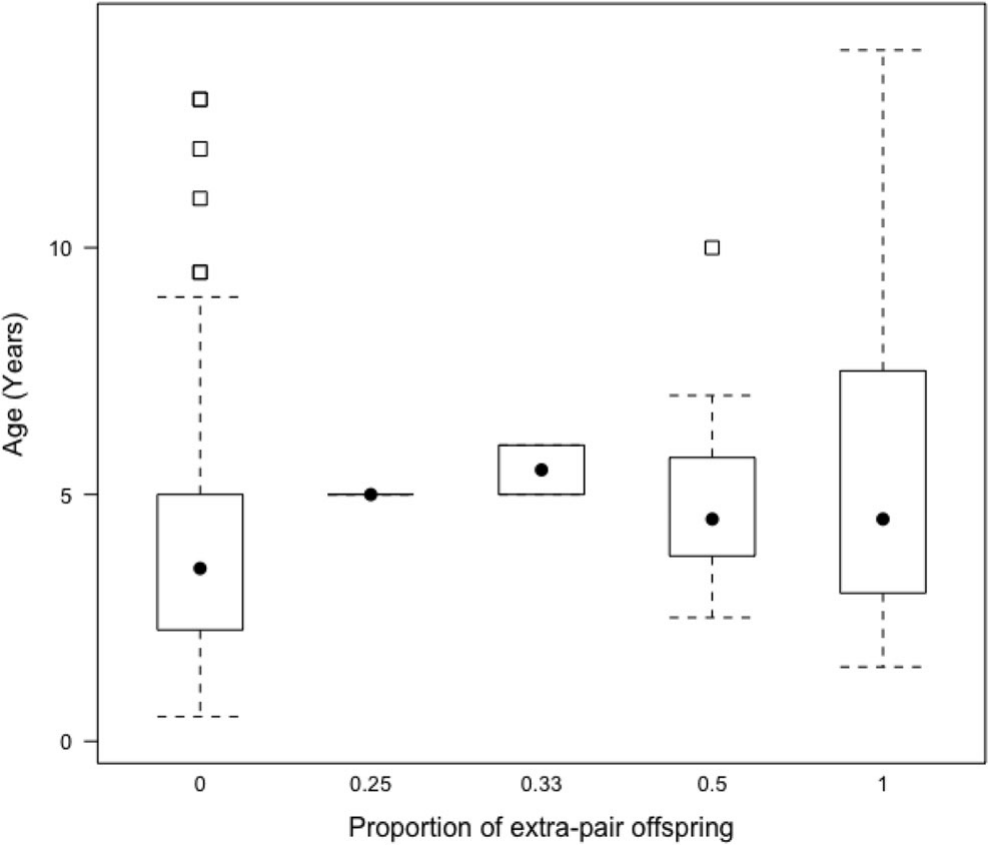
The proportion of EPO in the social brood (EPO/total brood size) with regard to age of the focal female. The black dot is the median age, lower and upper margin of the boxes represent the lower and upper quartiles (25 and 75%), dotted lines are whiskers (indicating variability outside the upper and lower quartiles) and black outlined squares are outliers

The total number of offspring (female *μ* = 1.15, SE = 0.03, male *μ* =1.29, SE = 0.04), the proportion of EPP and the propensity to have EPP, in a season were not associated with exploration of a novel environment (Figs S1–S6) or exploration of a novel object (Figs S7–S12) in males or females. There was an effect of age on whether the female had EPO in the social brood in a season, with the EPP rate increasing with age (*β* = 10.82, *pMCMC* = 0.01, Figs 1, S3, and *β* = 380.1, *pMCMC* = 0.002 Fig. S2, Fig. 2 ). The number of offspring in a season was positively correlated with the number of helpers (*β* = 0.26, *pMCMC* = 0.01, Fig. S1, *β* = 0.28, *pMCMC* = 0.01, Fig. S4, *β* = 0.28, I = 0.03, Fig. S7, and *β* = 0.29, *pMCMC* = 0.02, Fig. S10). Neither total offspring nor EPP in a season were significantly associated with year of offspring’s birth, insect abundance, sex, social status and the year of the focal individual’s birth (Figs 1, S1–S12).

### Pair similarity analyses

The total number of offspring in a season sired by either the male (*μ* = 0.93, SE = 0.06) or female (*μ* = 0.87, SE = 0.05) within the pair were not associated with the interaction between their own and their social partner’s exploration of a novel environment (Tables S1, S2), indicating that we found no significant differences in overall fitness. The total number of offspring in a season sired by a male increased with age (age: *β* = 3.08,* pMCMC* = 0.02 and age^2^: *β* = −2.68, *pMCMC* = 0.02, Table S2). The probability of a female’s offspring being sire by an extra-pair male in a season was higher in pairs that were mismatched in terms of exploration of the novel environment (*β* = − 6.81 e-5, *pMCMC* = 0.004, Tables 1, S3), but this effect was not seen for males (*β* = 2.03 e-5, *pMCMC* = 0.08, Table S4).

**Table 1:** Estimates of the posterior modes of the fixed effects in the binomial model for whether a female was assigned EPO: female/male year of birth, female/male exploration score and their interaction, female/male age (quadratic and linear terms), helper variable (the number of helpers in an offspring’s natal territory, divided by the total number of offspring gained in a season), annual insect abundance, year of offspring’s birth, male tent colour (*N*: blue = 52, green = 24) and female tent colour (*N*: blue = 48, green = 28, contrast level = blue), and male/female assay number. Posterior modes and associated 95% credible intervals, bold indicates effects for which the 95% credible interval does not overlap zero after FDR correction

	Posterior mode	Lower credible interval	Upper credible interval	*pMCMC*	FDR *pMCMC*
Female year of birth	− 0.115	− 0.494	0.312	0.536	
Female novel environment exploration score	− 0.007	− 0.075	0.052	0.869	
Male novel environment exploration score squared	0.001	− 0.001	0.002	0.023	0.080
Female novel environment exploration score*Male novel environment exploration score squared	**< − 0.001**	**< − 0.001**	**< −0.001**	**0.001**	**0.004**
Female age	0.496	− 6.27	7.86	0.883	
Female age squared	1.25	− 5.05	7.58	0.701	
Helper variable	− 1.48	− 3.17	0.316	0.087	
Insect abundance	0.764	− 0.696	2.25	0.310	
Year of offspring’s birth	0.178	− 0.297	0.631	0.419	
Male tent colour	− 0.719	− 4.87	2.93	0.728	
Female tent colour	0.843	− 2.32	4.12	0.592	
Male assay number	< 0.001	− 1.70	1.59	0.993	
Female assay number	1.56	− 0.304	3.90	0.074	
Male novel environment exploration assay year	0.619	− 1.25	2.53	0.472	
Female novel environment exploration assay year	− 0.201	− 1.86	1.62	0.783	

## Discussion

In this cooperatively breeding system, we have found no association between exploratory behaviours and the total number of offspring, the proportion of EPP and the propensity to have EPP in individuals. We also found no association between the number of offspring sired by a male or female in a pair and the interaction between the pair’s exploratory behaviours. However, we have found that the propensity for females to have EPO in the social brood was higher in pairs that were mismatched with regards to exploration of a novel environment, although the effect size was small, indicating that it is unlikely to be biologically important.

Behavioural similarity within the social pair has been shown to influence reproductive success (Dingemanse et al. 2004; Spoon et al. 2006; David et al. 2015). For example, in great tits (*Parus major*), males paired to dissimilar exploratory females provided less parental effort than males paired with similar females (David et al. 2015). Also in cockatiels (*Nymphicus hollandicus*), behavioural similarity within the social pair correlated with large clutch sizes, efficient coordination of incubation, and survival of chicks to independence (Spoon et al. 2006). Individuals in incompatible pairs may therefore counteract any potential reduction in the survival and future success of within-pair offspring if extra-pair mating provides fitness benefits (e.g. for females, indirect genetic effects, Mays and Hill 2004; Andersson and Simmons 2006; Wilson and Nussey 2010; and for males by increasing their reproductive success, e.g. Bateman 1948).

Genetically, variable traits, such as personality, are expected to persist in populations if balancing selection acts upon them over time (Dingemanse and Wolf 2010). The exploratory behaviour of individuals and the similarity of exploratory behaviour within the pair did not predict the total number of offspring they produced in a season, indicating no significant differences in overall fitness among personality types. We suggest that the null result is likely to have been caused by a trade-off between EPP and within-pair paternity for individuals, but we were unable to test this due to sample size. One of the mechanisms of balancing selection is frequency-dependent selection, where the fitness benefits of a reproductive strategy are related to the frequency with which it is expressed (Sinervo and Lively 1996). It has been postulated that frequency-dependent selection could maintain personality variation, and potentially the variation in pair exploratory behaviour that we have noted in this population (Dingemanse and Wolf 2010; Patrick et al. 2011).

Previous research in socially monogamous species has found that the exploratory or aggressive nature of an individual can influence the mode of paternity acquisition (While et al. 2009; Patrick et al. 2011; Martin et al. 2014). Therefore, further investigation in other species is warranted, particularly in a cooperatively breeding system. Barta (2016) postulates that inter-individual differences, such as personality, in the social environment create new behavioural alternatives and thus new selective forces. For example, it may pay to be choosier with regards to mate choice in a more phenotypically, and genetically, variable population. Understanding the association between EPP and exploratory behaviour in a cooperatively breeding species, such as the Seychelles warbler, can help us to understand why variation in EPP occurs and the role that personality may play.

We note that there are factors in our study that warrant future investigation. First, the effect we observed of higher EPO occurring in social broods mismatched for personality may be biologically relevant but small, and there could be several reasons for this. For example, the personalities of neighbouring individuals might suppress a male’s ability to sire extra-pair young. The incidence of EPP among individuals in a population can have a heritable component, as has been shown in female song sparrows (*Melospiza melodia*, *h*^2^ = 0.18; credible interval = 0.05–0.31, Reid et al. 2010); thus there can be genetic constraints on EPP in a mating system (Reid et al. 2010). We also do not know if the social partner has the potential to affect the expression of personality and the propensity of EPP in a focal individual, through indirect genetic effects (Niemelä and Santostefano 2015). Finally, in this study, we used wild birds that were held for a brief period in a captive environment, and we assumed that the exploratory behaviour that we measured correlated with actual activity and partner encounter rates. It is however debatable whether captive/laboratory based personality assays do (e.g. Herborn et al. 2010) or do not (e.g. Fisher et al. 2015) reflect behaviour in the wild, and it would be beneficial to confirm the association of exploratory behaviour with activity and encounter rates.

Although further study is needed using a larger dataset, we also found that the occurrence of cuckoldry by females increased at young ages. Age is an important determinant of parentage in male passerines (Griffith et al. 2002; Cleasby and Nakagawa 2012; Hsu et al. 2015), and there could be several reasons why we see this increase with age in young females. EPP may be constrained by parental care and thus, as females age, they might be better able to raise broods alone if cuckolded males reduce their parental care (Westneat et al. 1990; Gowaty 1996; Brouwer et al. 2017). If age indicates quality, then males may become more attractive to females as they age (Bouwman and Komdeur 2005). Females may become more selective about the males that sire their offspring as they age (Bouwman and Komdeur 2005). In the Seychelles warbler, EPP is regulated by mate guarding (Komdeur et al. 2007) and associated with the MHC diversity of the social partner (Richardson et al. 2005). It could therefore be that, as female Seychelles warblers age, they are more likely to have EPP in their brood due to a combination of choosiness increasing with age, and an increased ability to raise broods alone.

Age also determined the total number of offspring sired by a male in a season, with older males siring more offspring than their younger counterparts. In general, male reproductive success increases with age (Mauck et al*.* 2004; Willisch et al*.* 2012; Froy et al*.* 2013), and there may be several reasons for this. Older males may be preferred by females because they are experienced and are therefore able to provide a high level of parental care to within-pair offspring (Williams 1966; Trivers 1972; Forslund and Part 1995; Riechert et al. 2012). Older males may also be better at attracting mates and thus seek or gain EPP (Griffith et al. 2002). In the Seychelles warbler, it could be that older males sire a larger number of offspring due to a combination of increased ability to provide parental care and to attract potential mates.

To conclude, we have shown that there was no association between the number of offspring sired by a male or female in a pair and the interaction between the pair’s exploratory behaviour. We have also shown that the propensity for females to cuckold their mate (i.e. have EPP in their brood) is associated with pairs that are behaviourally disassortative for exploration of a novel environment. Overall, we conclude that, due to small effect size, the association between extra-pair paternity and exploratory behaviour is unlikely to be biologically relevant.

## Electronic supplementary material

ESM 1 (DOCX 466 kb)
