# Supplementary material for: Extra-pair parentage and personality in a cooperatively breeding bird
Source: Behav Ecol Sociobiol. 2018 Feb 15;72(3):37. doi: 10.1007/s00265-018-2448-z (PMC5814466; doi:10.1007/s00265-018-2448-z)
Supplement: Supplementary file 1 — (DOCX 466 kb) [file 265_2018_2448_MOESM1_ESM.docx]

**Supplementary materials**

**Behavioural Ecology and Sociobiology**

**Extra-pair parentage and personality in a cooperatively breeding bird**

Hannah A Edwards^1*^, Hannah L Dugdale^2,3^, David S Richardson^4,5^, Jan Komdeur^3^ and Terry Burke^1^

*^1^Department of Animal and Plant Sciences, University of Sheffield, Sheffield, S10 2TN, UK. +44 (0)114 222 0096*

*^2^School of Biology, The Faculty of Biological Sciences, University of Leeds, Leeds, LS2 9JT, UK. +44 (0)113 34 35598*

^3^*Behavioural Ecology and Physiological Group, Groningen Institute for Evolutionary Life Sciences, University of Groningen, PO Box 11103 9700 cc, Groningen, The Netherlands. +31 50 36 32056*

^4^ *School of Biological Sciences, University of East Anglia, Norwich Research Park, Norwich, NR4 7TJ, UK. +44 (0)1603 59 1496*

*^5^ Nature Seychelles, PO BOX 1310, Mahe, Republic of Seychelles*

*Corresponding author

Email: [*hannah.a.edwards@outlook.com*](mailto:bop12hae@sheffield.ac.uk) *(HAE)*

Telephone number: +44 (0)114 222 0112

**Supplementary Figure S1:** Forest plot of the novel environment exploration Poisson model of the total number of offspring a female was assigned parentage of

**Supplementary Figure S2:** Forest plot of the novel environment exploration binomial model of the proportion of extra pair offspring (EPO) to within pair offspring (WPO) assigned to a female

**Supplementary Figure S3:** Forest plot of the novel environment exploration binomial model for whether a female was assigned EPO

**Supplementary Figure S4:** Forest plot of the novel environment exploration Poisson model of the total number of offspring a male was assigned parentage of

**Supplementary Figure S5:** Forest plot of the novel environment exploration binomial model of the proportion of extra pair offspring (EPO) to within pair offspring (WPO) assigned to a male

**Supplementary Figure S6:** Forest plot of the novel environment exploration binomial model for whether a male was assigned EPO

**Supplementary Figure S7:** Forest plot of the novel object exploration Poisson model of the total number of offspring a female was assigned parentage of

**Supplementary Figure S8:** Forest plot of the novel object exploration binomial model of the proportion of extra pair offspring (EPO) to within pair offspring (WPO) assigned to a female

**Supplementary Figure S9:** Forest plot of the novel object exploration binomial model for whether a female was assigned EPO

**Supplementary Figure S10:** Forest plot of the novel object exploration Poisson model of the total number of offspring a male was assigned parentage of

**Supplementary Figure S11:** Forest plot of the novel object exploration binomial model of the proportion of extra pair offspring (EPO) to within pair offspring (WPO) assigned to a male

**Supplementary Figure S12:** Forest plot of the novel object exploration binomial model for whether a male was assigned EPO

**Supplementary Table S1:** Pair analysis-Table of the novel environment exploration Poisson model for total number of offspring the female was assigned parentage of

**Supplementary Table S2:** Pair analysis-Table of the novel environment exploration Poisson model for total number of offspring the male was assigned parentage of

**Supplementary Table S3:** Pair analysis-Table of the novel environment exploration binomial model for whether a female was assigned EPO

**Supplementary Table S4:** Pair analysis-Table of the novel environment exploration binomial model for whether a male was assigned EPO

**Individual analysis novel environment exploration**

**Figure S1**: Estimates of the posterior modes of the fixed effects in the Poisson model of the total number of offspring a female was assigned parentage of per season: year of birth, social status (N: only primary = 66, only non-primary = 8, assigned offspring as non-primary and as a primary = 11; contrast level = primary), novel environment exploration score, tent colour (N: blue = 67, green = 18; contrast level = blue), age (quadratic and linear terms), helper number (the number of helpers in an offspring’s natal territory), annual insect abundance, year of offspring’s birth and year of novel environment exploration assay. Posterior modes and associated 95% credible intervals (CrI), * indicates effects for which the 95% credible interval does not overlap zero after FDR correction.


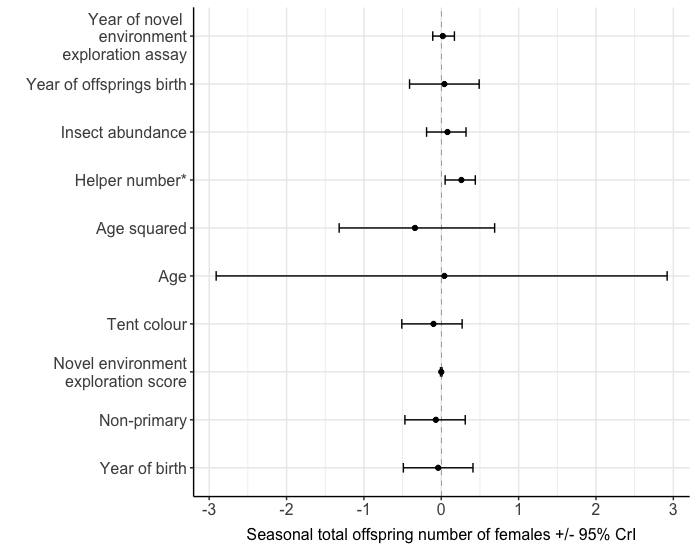


**Figure S2**: Estimates of the posterior modes of the fixed effects in the binomial model of the proportion of extra pair offspring (EPO) to within pair offspring (WPO) assigned to a female: year of birth, social status (N: only primary = 66, only non-primary = 8, assigned offspring as non-primary and as a primary = 11; contrast level = primary), novel environment exploration score, tent colour (N: blue = 135, green = 36; contrast level = blue), age (quadratic and linear terms), helper variable (the number of helpers in an offspring’s natal territory, divided by the total number of offspring gained in a season), annual insect abundance, year of offspring’s birth and year of novel environment exploration assay. Posterior modes and associated 95% credible intervals (CrI), * indicates effects for which the 95% credible interval does not overlap zero after FDR correction.


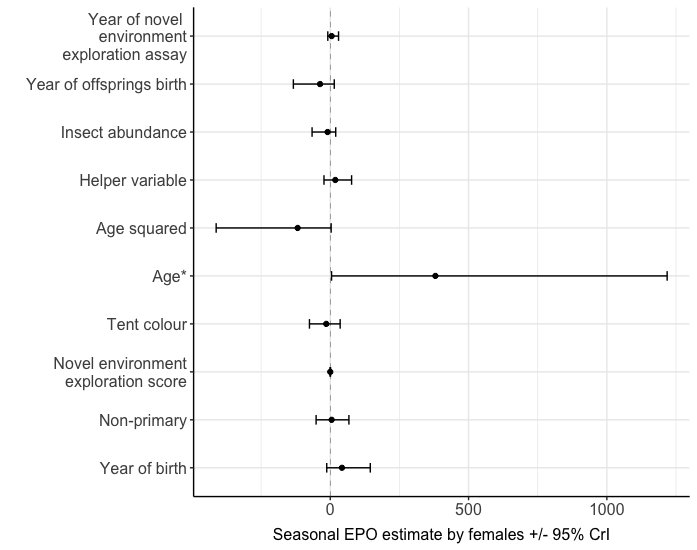


**Figure S3**: Estimates of the posterior modes of the fixed effects in the binomial model for whether a female was assigned EPO (yes/no): year of birth, social status (N: only primary = 66, only non-primary = 8, assigned offspring as non-primary and as a primary = 11; contrast level = primary), novel environment exploration score, tent colour (N: blue = 135, green = 36; contrast level = blue), age (quadratic and linear terms), helper variable (the number of helpers in an offspring’s natal territory, divided by the total number of offspring gained in a season), annual insect abundance, year of offspring’s birth and year of novel environment exploration assay. Posterior modes and associated 95% credible intervals (CrI), * indicates effects for which the 95% credible interval does not overlap zero after FDR correction.


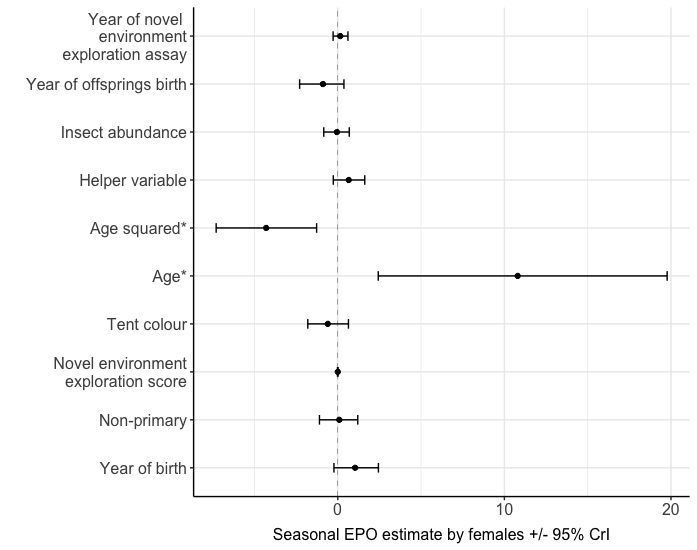


**Figure S4**: Estimates of the posterior modes of the fixed effects in the Poisson model of the total number of offspring a male was assigned parentage of per season: year of birth, social status (N: only primary = 84, assigned offspring as non-primary and as a primary = 2; contrast level = primary), novel environment exploration score, tent colour (N: blue = 68, green = 18; contrast level = blue), age (quadratic and linear terms), helper number (the number of helpers in an offspring’s natal territory), annual insect abundance, year of offspring’s birth and year of novel environment exploration assay. Posterior modes and associated 95% credible intervals (CrI), * indicates effects for which the 95% credible interval does not overlap zero after FDR correction.


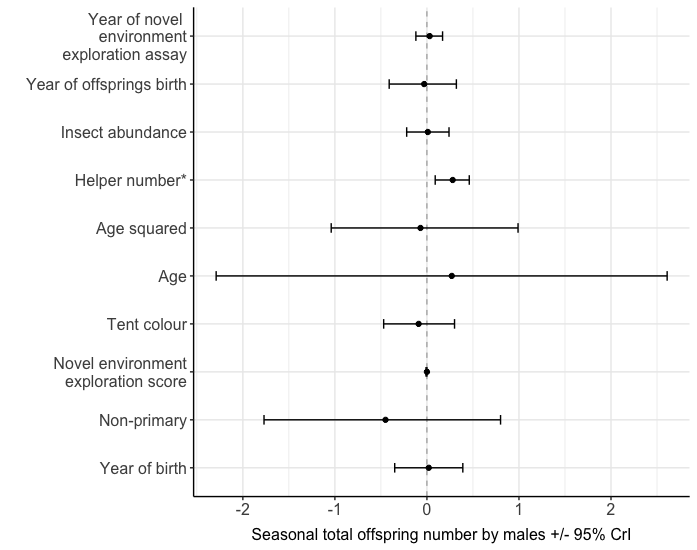


**Figure S5**: Estimates of the posterior modes of the fixed effects in the binomial model of the proportion of EPO to WPO assigned to a male: year of birth, social status (N: only primary = 84, assigned offspring as non-primary and as a primary = 2; contrast level = primary), novel environment exploration score, tent colour (N: blue = 68, green = 18; contrast level = blue), age (quadratic and linear terms), helper variable (the number of helpers in an offspring’s natal territory, divided by the total number of offspring gained in a season), annual insect abundance, year of offspring’s birth and year of novel environment exploration assay. Posterior modes and associated 95% credible intervals (CrI), * indicates effects for which the 95% credible interval does not overlap zero after FDR correction.


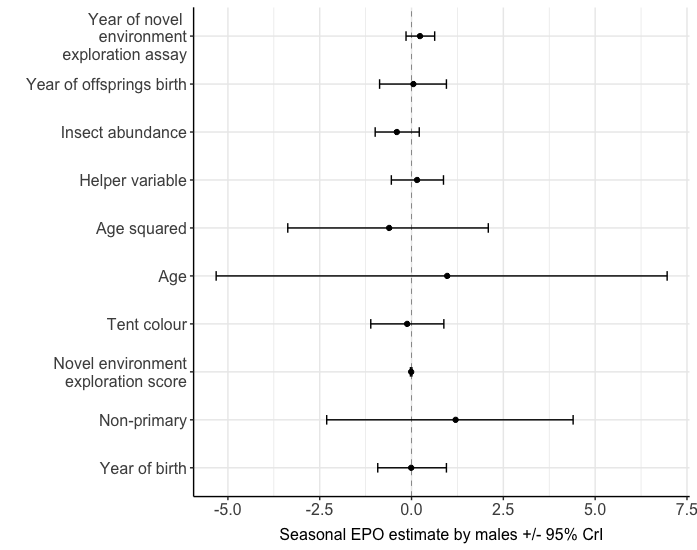


**Figure S6**: Estimates of the posterior modes of the fixed effects in the binomial model for whether a male was assigned EPO (yes/no): year of birth, social status (N: only primary = 84, assigned offspring as non-primary and as a primary = 2; contrast level = primary), novel environment exploration score, tent colour (N: blue = 68, green = 18; contrast level = blue), age (quadratic and linear terms), helper variable (the number of helpers in an offspring’s natal territory, divided by the total number of offspring gained in a season), annual insect abundance, year of offspring’s birth and year of novel environment exploration assay. Posterior modes and associated 95% credible intervals (CrI), * indicates effects for which the 95% credible interval does not overlap zero after FDR correction.


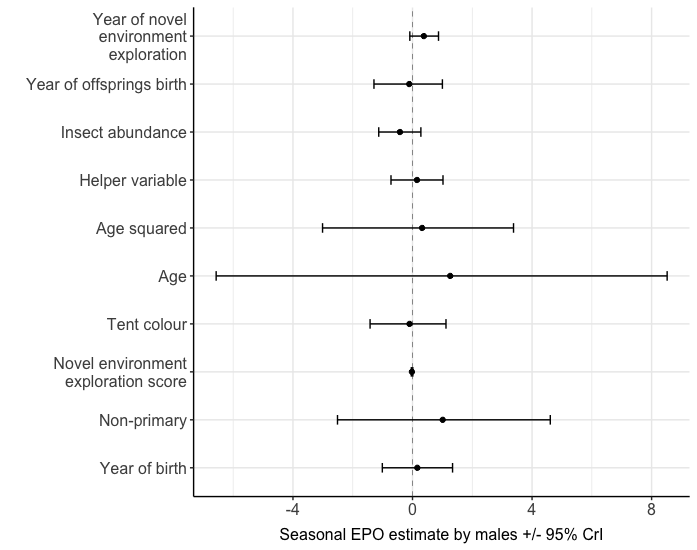


**Individual analysis novel object exploration**

**Figure S7**: Estimates of the posterior modes of the fixed effects in the Poisson model of the total number of offspring a female was assigned parentage of per season: year of birth, social status (N: only primary = 34, only non-primary = 4, assigned offspring as non-primary and as a primary = 5; contrast level = primary), novel object exploration score, age (quadratic and linear terms), helper number (the number of helpers in an offspring’s natal territory), annual insect abundance, year of offspring’s birth and year of novel object exploration assay. Posterior modes and associated 95% credible intervals (CrI), * indicates effects for which the 95% credible interval does not overlap zero after FDR correction.


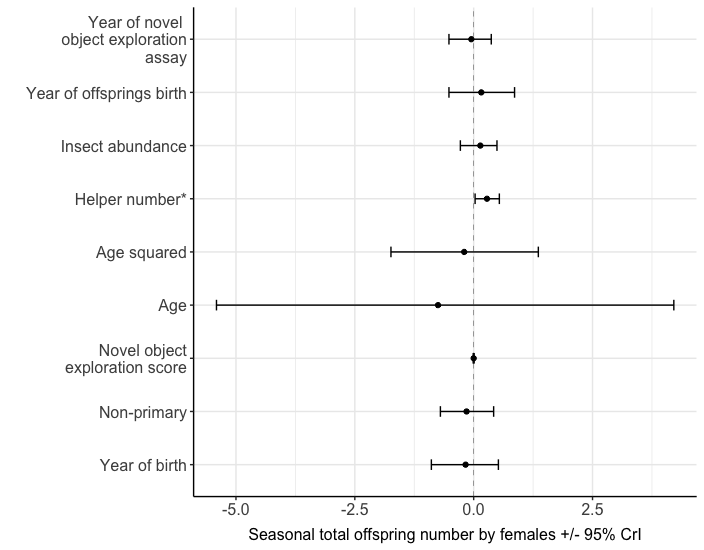


**Figure S8**: Estimates of the posterior modes of the fixed effects in the binomial model of the proportion of EPO to WPO assigned to a female: year of birth, social status (N: only primary = 34, only non-primary = 4, assigned offspring as non-primary and as a primary = 5; contrast level = primary), novel object exploration score, age (quadratic and linear terms), helper variable (the number of helpers in an offspring’s natal territory, divided by the total number of offspring gained in a season), annual insect abundance, year of offspring’s birth and year of novel object exploration assay. Posterior modes and associated 95% credible intervals (CrI), * indicates effects for which the 95% credible interval does not overlap zero after FDR correction.


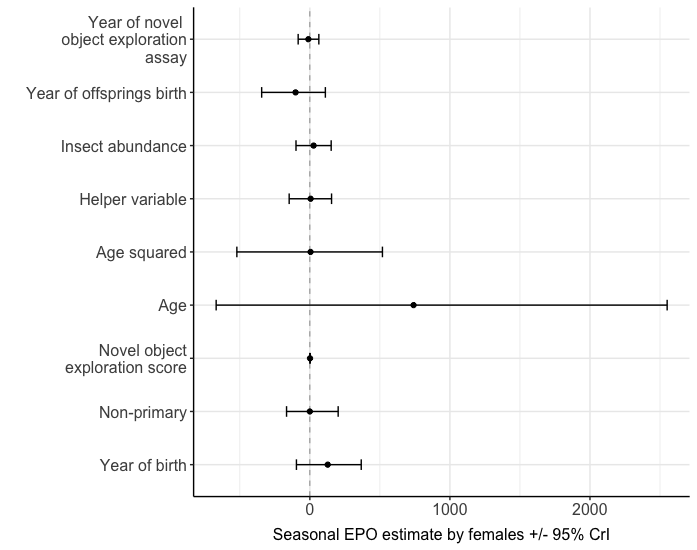


**Figure S9**: Estimates of the posterior modes of the fixed effects in the binomial model for whether a female was assigned EPO (yes/no): year of birth, social status (N: only primary = 34, only non-primary = 4, assigned offspring as non-primary and as a primary = 5; contrast level = primary), novel object exploration score, age (quadratic and linear terms), helper variable (the number of helpers in an offspring’s natal territory, divided by the total number of offspring gained in a season), annual insect abundance, year of offspring’s birth and year of novel object exploration assay. Posterior modes and associated 95% credible intervals (CrI), * indicates effects for which the 95% credible interval does not overlap zero after FDR correction.


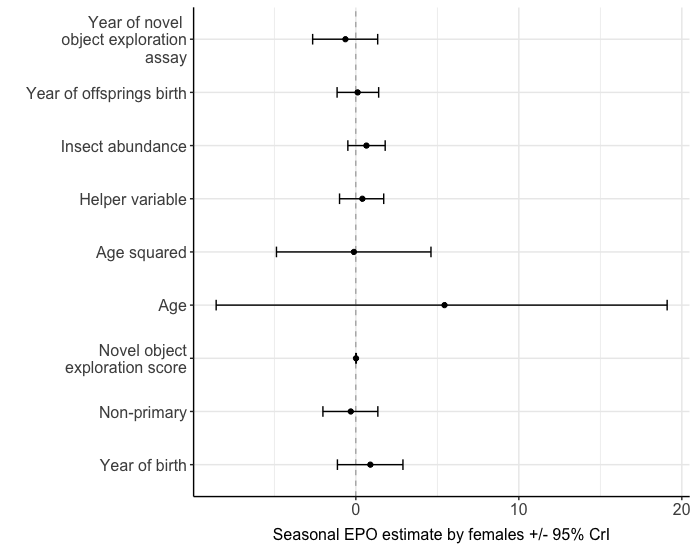


**Figure S10**: Estimates of the posterior modes of the fixed effects in the Poisson model of the total number of offspring a male was assigned parentage of per season: year of birth, novel object exploration score, age (quadratic and linear terms), helper number (the number of helpers in an offspring’s natal territory), annual insect abundance, year of offspring’s birth and year of novel object exploration assay. Posterior modes and associated 95% credible intervals (CrI), * indicates effects for which the 95% credible interval does not overlap zero after FDR correction.


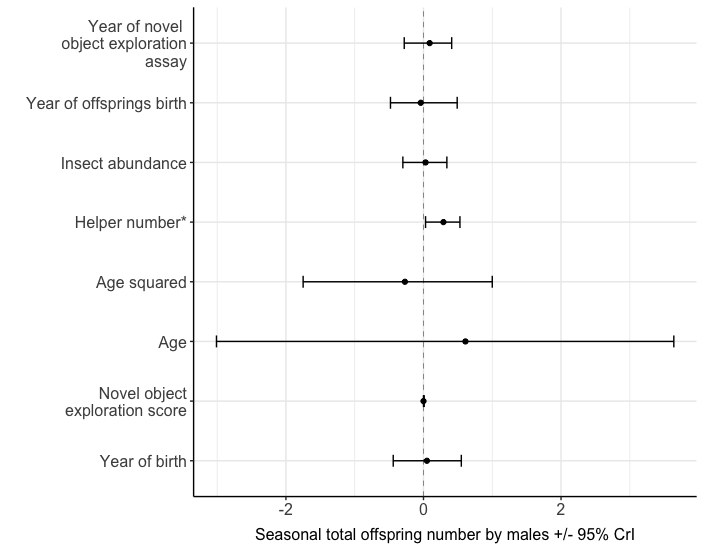


**Figure S11**: Estimates of the posterior modes of the fixed effects in the binomial model of the proportion of EPO to WPO assigned to a male: year of birth, novel object exploration score, age (quadratic and linear terms), helper variable (the number of helpers in an offspring’s natal territory, divided by the total number of offspring gained in a season), annual insect abundance, year of offspring’s birth and year of novel object exploration assay. Posterior modes and associated 95% credible intervals (CrI), * indicates effects for which the 95% credible interval does not overlap zero after FDR correction.


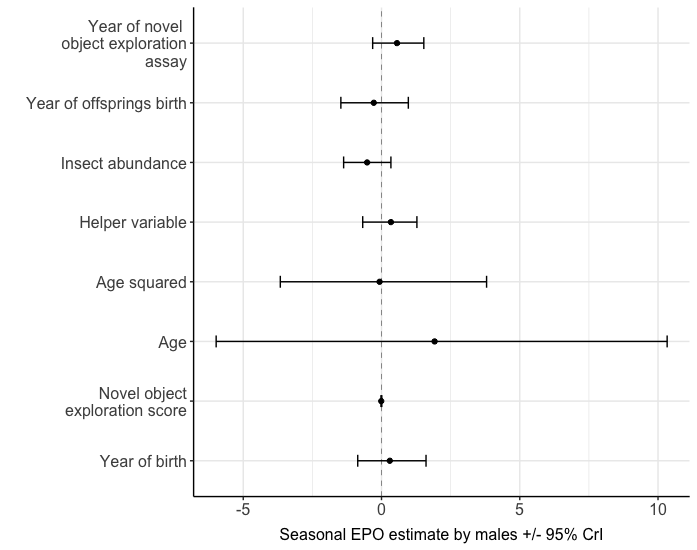


**Figure S12**: Estimates of the posterior modes of the fixed effects in the binomial model for whether a male was assigned EPO (yes/no): year of birth, novel object exploration score, age (quadratic and linear terms), helper variable (the number of helpers in an offspring’s natal territory, divided by the total number of offspring gained in a season), annual insect abundance, year of offspring’s birth and year of novel object exploration assay. Posterior modes and associated 95% credible intervals (CrI), * indicates effects for which the 95% credible interval does not overlap zero after FDR correction.


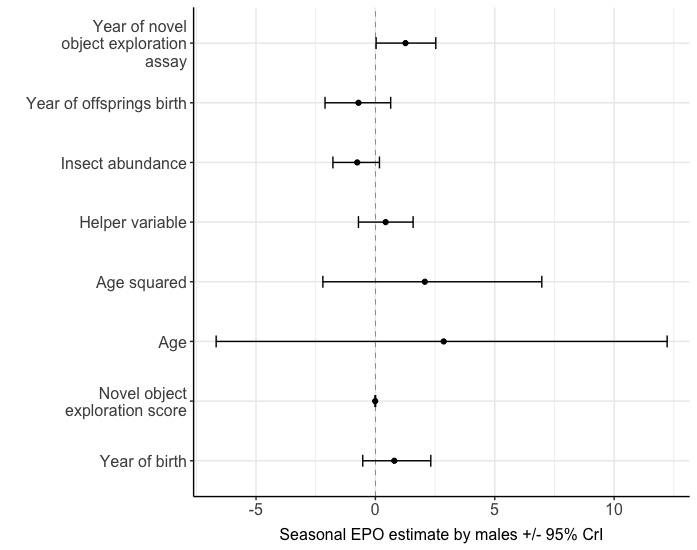


**Pair analysis novel environment exploration**

**Table S1**: Estimates of the posterior modes of the fixed effects in the Poisson model for total number of offspring the female was assigned parentage of: female/male year of birth, female/male exploration score and their interaction, female/male age (quadratic and linear terms), helper number (the number of helpers in an offspring’s natal territory), annual insect abundance, year of offspring’s birth, male tent colour (N: blue = 52, green = 24) and female tent colour (N: blue = 48, green = 28, contrast level = blue), male/female assay number and male/female novel environment exploration assay year. Posterior modes and associated 95% credible intervals (CrI), bold indicates effects for which the 95% credible interval does not overlap zero after FDR correction.

|  | **Posterior mode** | **Lower credible interval** | **Upper credible interval** | **pMCMC** |
| --- | --- | --- | --- | --- |
| Female year of birth | -3.89E-03 | -7.38E-02 | 5.64E-02 | 9.32E-01 |
| Male year of birth | -1.37E-03 | -5.22E-02 | 4.86E-02 | 9.47E-01 |
| Female novel environment exploration score | -3.26E-03 | -1.33E-02 | 7.36E-03 | 5.50E-01 |
| Male novel environment exploration score squared | 5.26E-05 | -7.68E-05 | 1.80E-04 | 4.15E-01 |
| Female novel environment exploration score*Male novel environment exploration score squared | -4.23E-06 | -1.16E-05 | 2.43E-06 | 2.21E-01 |
| Male age | -2.54E-01 | -2.17E+00 | 1.78E+00 | 7.77E-01 |
| Female age | 1.12E+00 | -8.88E-01 | 2.89E+00 | 2.69E-01 |
| Male age squared | 4.07E-01 | -1.34E+00 | 2.39E+00 | 6.44E-01 |
| Female age squared | -1.17E+00 | -3.01E+00 | 7.22E-01 | 2.20E-01 |
| Helper number | 2.84E-01 | -3.52E-02 | 6.05E-01 | 9.60E-02 |
| Insect abundance | 1.89E-01 | -2.07E-01 | 5.46E-01 | 3.17E-01 |
| Year of offspring’s birth | 4.70E-02 | -1.02E-01 | 1.74E-01 | 4.85E-01 |
| Male tent colour | -3.24E-01 | -9.48E-01 | 3.47E-01 | 3.18E-01 |
| Female tent colour | 1.30E-01 | -4.25E-01 | 6.87E-01 | 6.53E-01 |
| Male assay number | -4.87E-02 | -2.85E-01 | 2.14E-01 | 6.99E-01 |
| Female assay number | 2.16E-01 | -1.15E-01 | 5.05E-01 | 1.77E-01 |
| Male novel environment exploration assay year | 3.20E-02 | -2.96E-01 | 3.51E-01 | 8.43E-01 |
| Female novel environment exploration assay year | -6.57E-02 | -3.64E-01 | 2.40E-01 | 6.71E-01 |

**Table S2**: Estimates of the posterior modes of the fixed effects in the Poisson model for total number of offspring sired by the male: female/male year of birth, female/male exploration score and their interaction, female/male age (quadratic and linear terms), helper variable (the number of helpers in an offspring’s natal territory), annual insect abundance, year of offspring’s birth, male tent colour (N: blue = 52, green = 24), female tent colour (N: blue = 48, green = 28, contrast level = blue), male/female assay number and male/female novel environment exploration assay year. Posterior modes and associated 95% credible intervals (CrI), bold indicates effects for which the 95% credible interval does not overlap zero after FDR correction.

|  | **Posterior mode** | **Lower credible interval** | **Upper credible interval** | **pMCMC** | **FDR pMCMC** |
| --- | --- | --- | --- | --- | --- |
| Female year of birth | 2.97E-02 | -3.41E-02 | 9.02E-02 | 3.79E-01 | 5.00E-02 |
| Male year of birth | 5.29E-02 | 5.33E-03 | 9.87E-02 | 2.00E-02 |  |
| Female novel environment exploration score squared | -8.90E-05 | -2.09E-04 | 2.50E-05 | 1.41E-01 |  |
| Male novel environment exploration score | -1.01E-03 | -9.24E-03 | 7.65E-03 | 8.33E-01 |  |
| Female novel environment exploration score squared *Male novel environment exploration score | 1.78E-06 | -2.05E-06 | 5.41E-06 | 3.46E-01 |  |
| **Male age** | **3.08E+00** | **9.10E-01** | **5.16E+00** | **5.00E-03** | **1.50E-02** |
| Female age | 2.41E-01 | -1.64E+00 | 2.10E+00 | 8.08E-01 |  |
| **Male age squared** | **-2.68E+00** | **-4.72E+00** | **-4.97E-01** | **8.00E-03** | **2.40E-02** |
| Female age squared | -2.35E-01 | -1.91E+00 | 1.70E+00 | 8.07E-01 |  |
| **Helper number** | **5.15E-01** | **2.11E-01** | **8.34E-01** | **2.00E-03** | **8.00E-03** |
| Insect abundance | 4.61E-02 | -3.23E-01 | 4.38E-01 | 8.14E-01 |  |
| Year of offspring’s birth | -7.72E-03 | -1.39E-01 | 1.13E-01 | 8.77E-01 |  |
| Male tent colour | 2.08E-01 | -4.25E-01 | 8.37E-01 | 5.17E-01 |  |
| Female tent colour | -3.58E-01 | -8.84E-01 | 2.04E-01 | 2.07E-01 |  |
| Male assay number | 1.03E-01 | -1.39E-01 | 3.47E-01 | 4.40E-01 |  |
| Female assay number | 3.41E-02 | -3.15E-01 | 3.43E-01 | 8.17E-01 |  |
| Male novel environment exploration assay year | -2.38E-01 | -5.76E-01 | 7.84E-02 | 1.54E-01 |  |
| Female novel environment exploration assay year | 2.05E-01 | -1.04E-01 | 5.01E-01 | 1.79E-01 |  |

**Table S3**: Estimates of the posterior modes of the fixed effects in the binomial model for whether a female was assigned EPO: female/male year of birth, female/male exploration score and their interaction, female/male age (quadratic and linear terms), helper variable (the number of helpers in an offspring’s natal territory, divided by the total number of offspring gained in a season), annual insect abundance, year of offspring’s birth, male tent colour (N: blue = 52, green = 24) and female tent colour (N: blue = 48, green = 28, contrast level = blue), male/female assay number and male/female novel environment exploration assay year. Posterior modes and associated 95% credible intervals (CrI), bold indicates effects for which the 95% credible interval does not overlap zero after FDR correction.

|  | **Posterior mode** | **Lower credible interval** | **Upper credible interval** | **pMCMC** | **FDR pMCMC** |
| --- | --- | --- | --- | --- | --- |
| Female year of birth | -1.15E-01 | -4.94E-01 | 3.12E-01 | 5.36E-01 |  |
| Female novel environment exploration score | -7.16E-03 | -7.53E-02 | 5.25E-02 | 8.69E-01 |  |
| Male novel environment exploration score squared | 1.04E-03 | -3.84E-05 | 2.26E-03 | 2.31E-02 | 8.00E-02 |
| **Female novel environment exploration score*Male novel environment exploration score squared** | **-6.81E-05** | **-1.39E-04** | **-1.32E-05** | **1.00E-03** | **4.00E-03** |
| Female age | 4.96E-01 | -6.27E+00 | 7.86E+00 | 8.83E-01 |  |
| Female age squared | 1.25E+00 | -5.05E+00 | 7.58E+00 | 7.01E-01 |  |
| Helper variable | -1.48E+00 | -3.17E+00 | 3.16E-01 | 8.73E-02 |  |
| Insect abundance | 7.64E-01 | -6.96E-01 | 2.25E+00 | 3.10E-01 |  |
| Year of offspring’s birth | 1.78E-01 | -2.97E-01 | 6.31E-01 | 4.19E-01 |  |
| Male tent colour | -7.19E-01 | -4.87E+00 | 2.93E+00 | 7.28E-01 |  |
| Female tent colour | 8.43E-01 | -2.32E+00 | 4.12E+00 | 5.92E-01 |  |
| Male assay number | 3.43E-04 | -1.70E+00 | 1.59E+00 | 9.93E-01 |  |
| Female assay number | 1.56E+00 | -3.04E-01 | 3.90E+00 | 7.43E-02 |  |
| Male novel environment exploration assay year | 6.19E-01 | -1.25E+00 | 2.53E+00 | 4.72E-01 |  |
| Female novel environment exploration assay year | -2.01E-01 | -1.86E+00 | 1.62E+00 | 7.83E-01 |  |

**Table S4**: Estimates of the posterior modes of the fixed effects in the binomial model for whether a male was assigned EPO: female/male year of birth, female/male exploration score and their interaction, female/male age (quadratic and linear terms), helper variable (the number of helpers in an offspring’s natal territory, divided by the total number of offspring gained in a season), annual insect abundance, year of offspring’s birth, male tent colour (N: blue = 52, green = 24) and female tent colour (N: blue = 48, green = 28, contrast level = blue), male/female assay number and and male/female novel environment exploration assay year. Posterior modes and associated 95% credible intervals (CrI), bold indicates effects for which the 95% credible interval does not overlap zero after FDR correction.

|  | **Posterior mode** | **Lower credible interval** | **Upper credible interval** | **pMCMC** | **FDR pMCMC** |
| --- | --- | --- | --- | --- | --- |
| Male year of birth | 2.20E-01 | -1.42E-03 | 5.09E-01 | 2.91E-02 | 5.00E-02 |
| Female novel environment exploration score squared | -8.37E-05 | -6.55E-04 | 4.71E-04 | 7.25E-01 |  |
| Male novel environment exploration score | -1.23E-02 | -5.18E-02 | 2.69E-02 | 4.80E-01 |  |
| Female novel environment exploration score squared *Male novel environment exploration score | 2.03E-05 | -5.12E-06 | 5.39E-05 | 7.53E-02 |  |
| Male age | 6.79E+00 | 2.54E-01 | 1.44E+01 | 3.41E-02 | 4.50E-02 |
| Male age squared | -5.07E+00 | -1.13E+01 | 1.12E+00 | 8.53E-02 |  |
| Helper variable | 8.53E-02 | -1.36E+00 | 1.52E+00 | 8.94E-01 |  |
| Insect abundance | -6.94E-01 | -2.01E+00 | 4.48E-01 | 2.39E-01 |  |
| Year of offspring’s birth | -4.52E-02 | -5.63E-01 | 4.24E-01 | 9.28E-01 |  |
| Male tent colour | 1.93E+00 | -7.66E-01 | 4.98E+00 | 1.27E-01 |  |
| Female tent colour | -1.88E+00 | -4.72E+00 | 2.32E-01 | 8.94E-02 |  |
| Male assay number | 5.47E-01 | -7.94E-01 | 1.89E+00 | 3.45E-01 |  |
| Female assay number | -1.68E+00 | -3.60E+00 | 5.96E-02 | 3.11E-02 | 1.20E-01 |
| Male novel environment exploration assay year | -7.55E-01 | -2.39E+00 | 5.01E-01 | 2.72E-01 |  |
| Female novel environment exploration assay year | 1.03E+00 | -4.40E-01 | 2.68E+00 | 1.20E-01 |  |
